# How is weight teasing cross-sectionally and longitudinally associated with health behaviors and weight status among ethnically/racially and socioeconomically diverse young people?

**DOI:** 10.1186/s12966-022-01307-y

**Published:** 2022-06-23

**Authors:** Laura Hooper, Rebecca Puhl, Marla E. Eisenberg, Marla Reicks, Dianne Neumark-Sztainer

**Affiliations:** 1 Division of Epidemiology and Community Health, University of Minnesota, Suite 300, 1300 S, 2nd St., Minneapolis, MN 55454-1015 USA; 2 Department of Food Science and Nutrition, University of Minnesota, 225 Food Science and Nutrition, 1334 Eckles Ave, St. Paul, MN 55108 USA; 3 Department of Human Development & Family Sciences, College of Liberal Arts and Sciences, University of Connecticut, 348 Mansfield Road, U-1058, Storrs, CT 06269-1058 USA; 4 Division of General Pediatrics and Adolescent Health, Department of Pediatrics, University of Minnesota, 717 Delaware Street SE, 3rd Floor, Minneapolis, MN 55414 USA

**Keywords:** Weight teasing, Social stigma, Health behavior, Eating behavior, Dietary habits, Physical activity, Obesity, Body mass index, Adolescent, Young adult

## Abstract

**Background:**

Weight stigma is prevalent among young people and harmful to health. The current study used a health equity lens to examine cross-sectional and longitudinal associations between experiencing weight teasing (a form of weight stigma) with a range of weight-related health behaviors and weight status in an ethnically/racially and socioeconomically diverse sample of young people. We also assessed whether ethnicity/race and adolescent socioeconomic status (SES) operated as effect modifiers in these relationships.

**Methods:**

Adolescents (*n* = 1568) were enrolled in EAT 2010–2018 (Eating and Activity over Time) and followed into young adulthood. Weight teasing; screen time; moderate-to-vigorous physical activity (MVPA); sleep duration; breakfast frequency; fruit, vegetable, sugar-sweetened beverage (SSB), and fast-food intake; and body mass index (BMI) were assessed at baseline (mean age = 14.4 years) and eight-year follow-up (mean age = 22.2 years). Multivariate linear regression estimated marginal means and 95% confidence intervals. All analyses adjusted for BMI and sociodemographic characteristics.

**Results:**

Weight teasing was cross-sectionally associated with longer screen time, shorter sleep duration, and higher BMI during adolescence; and cross-sectionally associated with shorter sleep duration, lower breakfast frequency, higher fast-food intake, higher SSB intake, and higher BMI during young adulthood. In the longitudinal analyses, weight teasing was not associated with health behaviors but did predict higher BMI (teased: 28.2 kg/m^2^, not teased: 26.4 kg/m^2^, *p* < 0.001). White and higher adolescent SES subgroups had higher MVPA, more frequent breakfast intake, lower fast-food intake, and lower BMI than their respective counterparts. The relationships between weight teasing and health behaviors and weight status were largely consistent across ethnic/racial and adolescent SES subgroups.

**Conclusions:**

Findings add to growing evidence that weight-based mistreatment poses a threat to weight-related health and that young people across ethnic/racial and SES subgroups are vulnerable to the negative effects of weight teasing. Limitations include attrition at follow-up and the self-reported nature of many measures. Results suggest a need for increased attention to existing recommendations to reduce weight stigma in young people from diverse ethnic/racial and socioeconomic backgrounds including training for healthcare providers to better equip them to address the harms of weight teasing and foster more compassionate care to promote health-supporting behaviors in young people.

**Supplementary Information:**

The online version contains supplementary material available at 10.1186/s12966-022-01307-y.

## Background

Obesity is prevalent among adolescents and adults worldwide and is a risk factor for a number of chronic diseases such as cardiovascular disease, stroke, and type 2 diabetes [1–3]. In the United States (U.S.), obesity disproportionately affects Black, Indigenous, and other people of color (BIPOC) and people from low socioeconomic backgrounds, thus efforts to address this vital public health issue benefit from use of a health equity lens [4–6]. With the goal of improving health for the population as a whole, a health equity lens aims to identify and decrease health disparities in marginalized populations by acknowledging and attempting to correct the fact that research investigators and study participants often overrepresent privileged groups (e.g., white, middle- to upper-class) thus the conclusions and corresponding interventions might miss key factors important in meeting the health needs of underserved populations [4].

An important aspect of addressing weight-related health through a health equity lens is to focus on the negative social aspects of high body weight, namely societal weight stigma [7]. Weight stigma is based on social devaluation of people due to their high weight status or large body size, including stereotypes that these individuals lack willpower or discipline, are lazy, or are unmotivated [8]. This stigma manifests as prejudice and discrimination and can affect people across the weight spectrum. During adolescence and young adulthood, weight stigma most often occurs in the forms of teasing, bullying, and victimization [9], and is a common experience, with U.S. studies estimating prevalence in young people at 23–32% [10, 11]. Given the potential for intersectional impacts to young people who identify as BIPOC and those from low socioeconomic status (SES) households [12, 13], it is important to understand if the prevalence of weight-stigmatizing experiences differs by ethnicity/race or SES [14, 15] and how associations between weight stigma and weight-related health differ by these characteristics. Some studies have demonstrated no differences by ethnicity/race in the prevalence of weight stigma [13, 16], while other studies, including our prior work with this sample, have found a higher prevalence among BIPOC young people when compared to their white counterparts [15, 17]. Less work has focused on prevalence differences by SES [14], but our recent study found that weight teasing was more prevalent among young people from low SES backgrounds when compared to their higher SES counterparts [15]. Findings from this study were based on unadjusted analyses, given that the research goal was to describe sociodemographic differences in the prevalence of weight teasing.

To date, several studies in young people have demonstrated that experiencing weight stigma is a risk factor for poor psychosocial and behavioral health outcomes, such as higher prevalence of depressive symptoms [18–20], substance use [21], body dissatisfaction [18, 22], disordered eating behaviors [15, 17, 23–26], self-harm [18], social isolation [27], school avoidance [28], and low self-esteem [18, 20, 29, 30]. In addition, studies have found that weight stigma, above and beyond any effects of baseline weight status, is a risk factor for future weight gain [25, 31–34].

Despite evidence of associations between experiencing weight stigma and these adverse health outcomes, what remains more elusive is whether weight-stigmatizing experiences are a risk factor for weight-related health behaviors promoted by public health campaigns and monitored by healthcare providers, such as screen time; physical activity; sleep duration; and intake of breakfast, fruit, vegetables, sugar-sweetened beverages (SSB), and fast-food [35]. Few studies in young people have examined these relationships; in those that have, the focus has been on links between weight stigma and physical activity, yielding mixed results [36]. While some studies have reported associations between weight stigma and lower physical activity self-efficacy and motivation [19, 20, 22], physical activity avoidance [21, 28] and lower levels of physical fitness [20], other studies have reported null associations between weight stigma and physical activity levels [20, 22, 37] or mixed results [38]. One study in young people examined the relationship between weight stigma and sleep outcomes and found that the frequency of weight-based victimization at school and weight teasing from family members were both associated with self-reported difficulty falling asleep, although there were no associations with weight teasing from peers [38]. To our knowledge, no studies in adolescents and young adults have examined the relationship between weight stigma and screen time, breakfast intake, fruit intake, vegetable intake, SSB intake, or fast-food intake, although in adults, weight-stigmatizing experiences have been linked with higher energy intake [39] and higher intake of convenience foods such as fast-food and SSBs [40].

Furthermore, given structural inequities in the U.S., both BIPOC young people and young people from low SES backgrounds are disproportionately at risk for worse nutrition-, fitness-, and weight-related health outcomes [41, 42]. Socio-environmental contributions to these disparities include inequitable access to: 1) healthcare [5, 6], 2) opportunities to safely walk and play in one’s own neighborhood [43, 44], 3) developmentally-appropriate organized physical activities (i.e., dance, sports) [44], and 4) nutritious food [5, 41, 45]. Factors that play a role in inequitable access to nutritious food include high prevalence of fast-food and convenience stores in low-income, primarily BIPOC neighborhoods [5, 46], discriminatory farm subsidies [47, 48], targeted marketing of nutrient poor, energy dense foods to BIPOC communities [5, 46], and food policies that make these foods the accessible and affordable option [5, 45]. Because young people who have been marginalized by these environments may be vulnerable to intersectional forms of stigma (e.g. racism intersecting with weight stigma), it is important to elucidate the relationship between weight-based mistreatment and weight-related health to improve understanding of key factors involved in meeting the needs of these young people.

The present study begins to address these important gaps by examining associations between weight teasing and weight status and health behaviors in an ethnically/racially and socioeconomically diverse cohort of young people. We investigated whether weight teasing is associated with the outcomes of screen time, physical activity, sleep duration, and intake of breakfast, fruit, vegetables, SSBs, fast-food, and body mass index (BMI), cross-sectionally during both adolescence and young adulthood, and longitudinally over an eight-year follow-up. Additionally, we investigated whether health disparities are present in this sample, specifically whether our study’s outcomes are higher or lower based on ethnicity/race and adolescent SES and whether ethnicity/race and adolescent SES operate as effect modifiers in these relationships. We hypothesized that exposure to weight teasing would be associated with higher weight status in all three main effects analyses. We also hypothesized that participants who were BIPOC and from lower socioeconomic backgrounds would have outcomes considered to be less supportive of health when compared to their white and high SES counterparts. The other research questions had no a priori hypotheses and were considered exploratory given the mixed or limited literature on the main effects associations between weight-stigmatizing experiences and these health behaviors and given the paucity of literature examining whether ethnicity/race and SES operate as effect modifiers in these relationships. Investigating these underexamined questions can help improve understanding of the weight stigma-health behavior relationship among young people from diverse backgrounds, help clarify how weight stigma contributes to adverse health outcomes, and point to specific areas of focus for future research.

## Methods

### Study design and participants

Data were collected as part of EAT 2010–2018 (Eating and Activity over Time), a population-based, longitudinal study designed to examine dietary intake, physical activity, weight control behaviors, weight status, and factors associated with these outcomes in young people [49]. For EAT 2010, the study population included adolescents from 20 public middle and high schools in the urban area of Minneapolis/St. Paul, in the State of Minnesota, in the U.S. Adolescents completed classroom surveys and anthropometric measures at school [50]. The follow-up EAT 2018 assessment was designed to examine changes in weight-related outcomes as participants progressed through adolescence and into young adulthood. Participants completed the baseline EAT 2010 study as adolescents during the 2009–2010 academic year and completed the follow-up EAT 2018 survey as young adults in 2017–2018.

Of the original 2793 participants, 410 (14.7%) were lost to follow-up for various reasons, primarily missing contact information at EAT 2010 or no address found at follow-up (*n* = 397). Invitations to participate in the online EAT 2018 survey were mailed to the remaining 2383 young people. To encourage participation, non-responders were mailed up to eight reminders. Additional attempts were made to contact young people using email, phone calls, text messages, messaging through social media, and home visits. The University of Minnesota’s Institutional Review Board Human Subjects Committee approved all study protocols.

### Survey development

For the EAT 2010 survey, a 235-item self-report instrument, test–retest reliability of measures and internal consistency of survey items were assessed in a separate sample of 129 middle and high school students over a one-week period [50]. To allow for longitudinal comparisons, key items from the EAT 2010 survey were retained on the follow-up EAT 2018 survey [49]. For EAT 2018, test–retest reliability of measures was assessed in a subgroup of 112 young adult participants over a three-week period. A semi-quantitative Food Frequency Questionnaire (FFQ) was administered with the EAT 2018 survey to assess usual past year intake of fruit, vegetables, SSBs, and other food groups. Previous studies have examined and reported on the reliability and validity of intake estimates [51–53]. At baseline (EAT 2010), the 152-item youth version of the FFQ, the Young Adult Food Frequency Questionnaire (YAQ), was used to assess dietary intake; it has undergone extensive testing for validity and reproducibility in adolescents [54, 55]. For both the FFQ and the YAQ, participant responses were excluded if they reported a biologically implausible level of total energy intake (< 500 kcal/day or > 5000 kcal/day) or left 20 or more items blank (4.4% at baseline, 11.1% at follow-up).

### Primary measures

#### Weight teasing

Weight teasing was assessed at baseline and follow-up using the identical question, “How often do any of the following things happen? ….. You are teased about your weight.” Responses included “never, less than once a year, a few times a year, a few times a month, and at least once a week” [56]. This variable was dichotomized with “never” coded as “no” and all other response options coded as “yes” (test‐retest *r* = 0.73). Dichotomous coding was used because previous studies in young people have shown that reports of ever experiencing weight teasing (versus never) are predictive of adverse health outcomes [17, 18, 25]. At baseline, 2.7% were missing this variable; 2.8% were missing it at follow-up.

#### Screen time

To assess screen time at baseline, participants were asked “In your free time on an average weekday….how many hours do you spend doing the following activities?” Activities included “watching TV/DVDs/videos; using a computer (not for homework); Xbox/Play-station/other electronic games that you play while sitting.” Due to secular changes in device use among the population of young people, screen time at follow-up was assessed with the following question, “On an average weekday…how many hours of recreational screen time (for example, television, computer, social media, video games, smartphone or tablet) do you have a day? Do not include activities you do for work or school.” At both timepoints, the same question was additionally asked for an average weekend day; seven response options ranged from 0 to 5 + hours for each question; and weekly screen time hours were calculated from a weighted mean [57]. Test–retest reliability was *r* = 0.86 (baseline) and *r* = 0.76 (follow-up). At baseline, 0.1% were missing this variable; 1.2% were missing it at follow-up.

#### Physical activity

The modified Godin-Shephard Leisure-Time Exercise Questionnaire recall was used to assess hours of past-week exercise at both baseline and follow-up [58, 59]. Each item included relevant exercise examples for strenuous exercise (aerobics, basketball), moderate exercise (skiing, dancing), and mild exercise (bowling, golf) with six response options ranging from “none” to “6 + hours a week.” The midpoint of each response was used to calculate the sum of moderate-to-vigorous physical activity (MVPA) hours per week [60]. Test–retest reliability was *r* = 0.85. At baseline, 0.2% were missing this variable; 0.5% were missing it at follow-up.

#### Sleep duration

Sleep duration was assessed by asking, “On an average weekday (Monday-Friday), what time do you go to bed (to go to sleep)? What time do you get out of bed (to start your day)?” These questions were repeated for the weekend, and the same method was used to assess sleep duration at baseline and follow-up. Hours per night were averaged across weekday and weekend days (range: 4–16) [61]. Test–retest reliability was *r* = 0.56. At baseline, 6.9% were missing this variable; 9.7% were missing it at follow-up.

#### Breakfast intake

Frequency of breakfast intake was assessed at both baseline and follow-up by asking, “During the past week, how many days did you eat breakfast?” Response options were “never, 1–2 days, 3–4 days, 5–6 days, and every day” [62]. They were recoded as number of days per week (range: 0–7, test–retest *r* = 0.76). At baseline, 0.2% were missing this variable; 0.6% were missing it at follow-up.

#### Fruit and vegetable intake

Fruit and vegetable intake were assessed using the YAQ at baseline and the FFQ at follow-up. A daily serving was defined as the equivalent of one-half cup for fruit and vegetables, and vegetables excluded potatoes. Daily servings of fruit and vegetables were summed separately (range_fruit_: 0–10, range_vegetables_: 0–8). For fruit intake, 1.5% were missing this variable at baseline and 5.2% at follow-up. For vegetable intake, 1.5% were missing this variable at baseline and 8.5% at follow-up.

#### Sugar-sweetened beverage intake

As a part of the YAQ at baseline and the FFQ at follow-up, participants were asked to report their intake over the past year of regular (i.e., non-diet) soda. Responses ranged from “never / < 1 glass per month” to “ ≥ 3 glasses per day.” On the EAT 2010 and 2018 surveys, participants were asked, “In the past year, how many times did you usually drink….an energy drink (such as Red Bull, Full Throttle, Rockstar, etc.)….a sports drink (such as Gatorade, Powerade, etc.)?” Response options ranged from “never or less than once per month” to “2 or more per day.” A serving was defined as the equivalent of one glass, bottle, or can. Regular soda, sports drinks, and energy drinks were aggregated to create the SSB variable (range 0–28 servings per week) [63]. At baseline, 0.5% were missing this variable; 6.4% were missing it at follow-up.

#### Fast-food intake

At both baseline and follow-up, participants reported fast-food intake by answering the following question, “In the past month, how often did you eat something from the following types of restaurants… (1) traditional ‘burger-and-fries’ fast-food restaurant…(2) Mexican fast-food restaurant…(3) fried chicken…(4) sandwich or sub shop…(5) pizza place.” Response options included “never/rarely, 1–3 times per month, 1–2 times per week, 3–4 times per week, 5–6 times per week, and ≥ 1 times per day.” Data were aggregated to create one continuous variable for fast-food intake (range: 0–94 times per month; test–retest *r* = 0.73). At baseline, 0.3% were missing this variable, and 0.3% were excluded for reporting implausible values. At follow-up, 0.6% were missing this variable, and 0.6% were excluded for reporting implausible values.

#### Weight status

At baseline, adolescent weight and height were measured by trained staff in a private area, using standardized procedures [64]. Weight status at follow-up was based on self-reported weight and height, which highly correlate with objectively measured weight and height in young people (*r* = 0.88 for males and 0.85 for females) [65]. BMI-for-age (percentile) at baseline and BMI (kg/m^2^) at follow-up were derived from weight and height using the Centers for Disease Control and Prevention guidelines [66]. At baseline, 1.7% were missing this variable; 2.7% were missing it at follow-up.

### Sociodemographic measures

#### Adolescent socioeconomic status

Five categories of adolescent SES were generated using classification tree methodology [67, 68]. Highest parent/guardian education level primarily determined SES in adolescence. Family eligibility for free/reduced price lunch, family receipt of public assistance, and parent/guardian employment status were subsidiary variables in estimating adolescent SES. SES assessed during adolescence was used for all analyses because evidence suggests that low SES and other adversities during childhood and adolescence are good predictors of poor health outcomes during adulthood [69–72].

#### Gender/sex

Gender/sex was assessed at follow-up with the following question, “Are you….? Male, Female, Different identity (please specify): ____.” For simplicity, gender/sex is referred to as gender throughout the remainder of this article.

#### Other sociodemographic characteristics

Age and ethnicity/race were self-reported by young people. Ethnicity/race was assessed at baseline with the following question: “Do you think of yourself as…? White, Black or African American, Hispanic or Latino, Asian American, Native Hawaiian or Pacific Islander, American Indian or Native American, or Other: ____.” Due to small numbers, Native Hawaiian or Pacific Islander, American Indian or Native American, and Other were coded together as “Mixed or Other Race” [50, 67]. Test–retest agreement was 98–100%.

### Statistical analysis

Descriptive statistics including frequencies and percentages of ethnicity/race, adolescent SES, and gender, and mean and standard deviation of age, BMI, and weight teasing status were summarized. Multivariate linear regression, adjusted for ethnicity/race, adolescent SES, gender, and BMI, examined the relationship between weight teasing and outcomes (screen time; MVPA; sleep duration; intake of breakfast, fruit, vegetables, SSB, fast-food; and BMI) to estimate marginal means and 95% confidence intervals, cross-sectionally at baseline (EAT 2010), cross-sectionally at follow-up (EAT 2018), and longitudinally (EAT 2010–2018). Longitudinal models were additionally adjusted for the outcome assessed at baseline. We regard the potential confounders of ethnicity/race, adolescent SES, gender, and BMI as proxies for exposure to structural racism, classism, sexism, and weight stigma respectively [5, 15, 73]. These proxies are considered imperfect because of the heterogeneity of experiences within oppressed groups and the innate limitations that come with the categorization of these demographic characteristics [15, 74, 75]. There were no observed associations between age and the exposure variable weight teasing. Therefore, age was not included as a covariate in the statistical models. A sensitivity rank regression analysis was conducted for all variables that were not normally distributed (screen time and intake of fruit, vegetable, SSB, and fast-food).

ANOVA tests examined differences in the means of the health behaviors and BMI by ethnicity/race and adolescent SES. To investigate whether ethnicity/race and adolescent SES operate as effect modifiers in the relationship between weight teasing and the outcomes (health behaviors and weight status), interaction terms between ethnicity/race and weight teasing and between adolescent SES and weight teasing were added to each cross-sectional and longitudinal regression model (separate models). Stratified models were additionally run when an interaction term was statistically significant (*p* < 0.1). For ANOVA tests and assessment of effect modification, to assist with interpretability of findings, a 3-category adolescent SES variable was created – low, middle (low middle combined with middle), and high (upper middle combined with high). For all analyses, Cohen’s d statistic was used to calculate effect sizes to aid in practical interpretation of results with the following interpretation: small (d = 0.2), medium (d = 0.5), large (d = 0.8) [76].

The sample of young adults who completed surveys at both timepoints included 65.8% of the original participants for whom contact information was available at EAT 2018. In addition, the analytic sample was more likely than the baseline sample to have parents/guardians with low educational attainment, identify as BIPOC, and report being born outside the U.S. when compared to responders. Therefore, inverse probability weighting was used for all analyses [77, 78]. Inverse probability weighting minimizes potential response bias due to missing data and allows for extrapolation back to the original school-based EAT 2010 sample. Inverse probability weights were derived as the inverse of the estimated probability that an individual responded at both timepoints based on several characteristics reported in 2010, including parental educational attainment, ethnicity/race, nativity, past year frequency of dieting, and weight status. To be included in the analytic sample, participants needed to have completed both EAT 2010 and EAT 2018 surveys (*n* = 1568) and needed data for the weight teasing exposure, the outcome (health behavior or weight status), and the covariates; thus the analytic sample varied slightly for each analysis. All analyses were conducted using SAS 9.4 (Cary, NC, copyright 2015).

## Results

### Participant characteristics

Weight teasing was prevalent in this sample with 34.1% of participants reporting being teased about their weight during adolescence and 41.5% during young adulthood. The ethnic/racial distribution of the sample included 28.6% African American/Black, 20.0% Asian American, 19.1% white, 17.2% Latinx/Hispanic, and 15.2% mixed/other (Table 1). Most of the Asian American participants reported Southeast Asian heritage; approximately 80.6% of this group were Hmong. For adolescent SES, the distribution of the sample was 39.5% low, 22.2% low middle, 17.6% middle, 13.2% upper-middle, and 7.5% high. Distribution by gender was 53.6% female, 45.8% male, and 0.6% different identity (e.g., transgender, non-binary). Mean age was 14.4 ± 2.0 years at baseline and 22.2 ± 2.0 years at follow-up. Mean BMI-for-age was 69.2 percentile during adolescence (EAT 2010) and 27.2 kg/m^2^ during young adulthood (EAT 2018). There were no significant differences in parental education, nativity status, and ethnicity/race between the weighted analytic sample and the full EAT 2010 sample (*p* > 0.9); all reported findings reflect weighted analyses.

**Table 1:** Sociodemographic characteristics, weight status, and exposure to weight teasing among young people (*N* = 1568) in Minneapolis-St Paul, Minnesota at baseline (EAT 2010) and at eight-year follow up (EAT 2018)

Characteristics	Mean ± SD or % (n)
Ethnicity / Race (%)^a^	
Asian American	20.0 (306)
Black/African American	28.6 (437)
Latinx/Hispanic	17.2 (263)
Mixed/Other Race	15.2 (232)
White	19.1 (292)
Socioeconomic Status (%)^a^	
Low	39.5 (588)
Low Middle	22.2 (331)
Middle	17.6 (262)
Upper Middle	13.2 (196)
High	7.5 (112)
Gender (%)^b^	
Female	53.6 (813)
Male	45.8 (696)
Different identity	0.6 (9)
Age (mean years)	14.4 ± 2.0^a^
	22.2 ± 2.0^b^
BMI-for-age (percentile)	69.2 ± 27.8^a^
BMI (kg/m^2^)	27.2 ± 7.0^b^
Weight Teasing (any)	34.1 (523)^a^
	41.5 (618)^b^

^a^Assessed at baseline (EAT 2010)

^b^Assessed at follow up (EAT 2018)

### Associations between weight teasing and health behaviors: main effects

At baseline, after accounting for ethnicity/race, adolescent SES, gender, and weight status, weight teasing was associated with higher screen time, shorter sleep duration, and higher vegetable intake (Table 2). For example, among adolescents teased about their weight, mean screen time was 42.6 h per week, compared to 38.9 h per week among those not teased (d = 0.13, *p* = 0.011). At follow-up, weight teasing was cross-sectionally associated with shorter sleep duration, less frequent breakfast intake, higher SSB intake, and higher fast-food intake, in adjusted models (Table 2). For example, among young adults teased about their weight, mean fast-food intake was 16.7 times per month, compared to 13.2 times per month among those not teased (d = 0.24, *p* < 0.001). In longitudinal analyses, all health behaviors were unrelated to baseline weight teasing (Table 3). The sensitivity rank regression analysis showed similar results to the linear regression analysis for all results.

**Table 2:** Cross-sectional relationships of health behaviors and weight status by weight teasing status in young people, effect sizes and marginal means, main effects models

**Adolescence (EAT 2010)**				
**Outcome**	**Cohen’s d Statistic**	**Marginal Mean (95% CI)**		***p-value***
		Not Teased	Teased	
Screen Time (hours/week)	0.13	38.9 (37.3, 40.5)	42.6 (40.3, 44.8)	**0.011**
MVPA (hours/week)	0.06	5.9 (5.6, 6.2)	5.6 (5.2, 6.0)	0.220
Sleep duration (hours/night)	0.12	8.6 (8.5, 8.6)	8.4 (8.3, 8.5)	**0.026**
Breakfast intake (days/week)	0.10	4.4 (4.2, 4.5)	4.1 (3.9, 4.3)	0.067
Fruit intake (servings/day)	0.07	2.1 (2.0, 2.2)	2.2 (2.1, 2.4)	0.160
Vegetable intake (servings/day)	0.19	1.2 (1.2, 1.3)	1.5 (1.4, 1.6)	** < 0.001**
(SSB intake servings/week)	0.03	5.3 (4.9, 5.7)	5.5 (5.0, 6.0)	0.576
Fast-Food intake (times/month)	0.07	12.5 (11.6, 13.4)	13.6 (12.3, 14.9)	0.172
BMI-for-age (percentile)	0.31	66.1 (64.4, 67.8)	75.0 (72.6, 77.4)	** < 0.001**
**Young Adulthood (EAT 2018)**				
**Outcome**	**Cohen’s d Statistic**	**Marginal Mean (95% CI)**		***p***-**value**
		**Not Teased**	**Teased**	
Screen Time (hours/week)	0.10	24.8 (24.0, 25.6)	26.0 (25.0, 26.9)	0.068
MVPA (hours/week)	0.07	4.6 (4.3, 4.8)	4.3 (4.0, 4.6)	0.205
Sleep duration (hours/night)	0.24	8.6 (8.5, 8.7)	8.3 (8.1, 8.4)	** < 0.001**
Breakfast intake (days/week)	0.13	3.9 (3.7, 4.0)	3.5 (3.3, 3.7)	**0.011**
Fruit intake (servings/day)	0.03	2.0 (1.9, 2.1)	1.9 (1.8, 2.1)	0.634
Vegetable intake (servings/day)	0.07	2.2 (2.1, 2.3)	2.1 (1.9, 2.2)	0.206
SSB intake (servings/week)	0.12	3.4 (3.1, 3.8)	4.1 (3.6, 4.5)	**0.031**
Fast-Food intake (times/month)	0.24	13.2 (12.2, 14.1)	16.7 (15.5, 17.8)	** < 0.001**
BMI (kg/m^2^)	0.47	25.8 (25.4, 26.3)	29.0 (28.5, 29.6)	** < 0.001**

BMI-for-age and BMI models adjusted for ethnicity/race, socioeconomic status, and gender. All other models adjusted for ethnicity/race, socioeconomic status, gender, and weight status. Gender assessed at follow-up; other covariates assessed at baseline

Sample size range: 1190–1479; Weighted analyses

Bold values indicate statistical significance at *p* < 0.05

*MVPA* Moderate-to-vigorous physical activity, *SSB* Sugar-sweetened beverage, *BMI* Body mass index

**Table 3:** Longitudinal relationships of health behaviors and weight status at 8-year follow-up by weight teasing status at baseline, effect sizes and marginal means (EAT 2010–2018, from adolescence to young adulthood), main effects models

Outcome	Cohen’s d Statistic	Marginal Mean (95% CI)		*p*-value
		Not Teased	Teased	
Screen Time (hours/week)	0.00	25.2 (24.5, 26.0)	25.1 (24.0, 26.2)	0.838
MVPA (hours/week)	0.03	4.4 (4.2, 4.7)	4.5 (4.2, 4.9)	0.582
Sleep duration (hours/night)	0.08	8.5 (8.4, 8.6)	8.4 (8.2, 8.5)	0.127
Breakfast intake (days/week)	0.04	3.7 (3.5, 3.8)	3.8 (3.6, 4.0)	0.453
Fruit intake (servings/day)	0.04	2.0 (1.8, 2.1)	2.0 (1.8, 2.2)	0.508
Vegetable intake (servings/day)	0.02	2.2 (2.0, 2.3)	2.2 (2.0, 2.4)	0.791
SSB intake (servings/week)	0.03	3.6 (3.2, 3.9)	3.8 (3.3, 4.2)	0.552
Fast-Food intake (times/month)	0.03	14.4 (13.6, 15.3)	14.9 (13.7, 16.2)	0.535
BMI (kg/m^2^)	0.31	26.4 (26.1, 26.8)	28.2 (27.8, 28.7)	** < 0.001**

Models adjusted for ethnicity/race, socioeconomic status, gender, outcome assessed at baseline, and weight status

Gender assessed at follow-up; other covariates assessed at baseline

Sample size range: 1130–1475; Weighted analyses

Bold values indicate statistical significance at *p* < 0.05

*MVPA* Moderate-to-vigorous physical activity, *SSB* Sugar-sweetened beverage, *BMI* Body mass index

### Associations between weight teasing and weight status: main effects

Weight teasing was associated with higher BMI-for-age in the cross-sectional analysis during adolescence (not teased: 66.1 percentile, teased: 75.0 percentile, d = 0.31, *p* < 0.001), the cross-sectional analysis during young adulthood (not teased: 25.8 kg/m^2^, teased: 29.0 kg/m^2^, d = 0.47, *p* < 0.001), and the longitudinal analysis (not teased: 26.4 kg/m^2^, teased: 28.2 kg/m^2^, d = 0.31, *p* < 0.001), after accounting for sociodemographic characteristics and, in the longitudinal analysis, baseline weight status (Tables 2 and 3). These results did not represent a difference in weight status categories, as both the teased and not teased groups had a mean BMI-for-age that fell into the same category (5th percentile to less than the 85th percentile) for cross-sectional analysis during adolescence, and a mean BMI in the same category (25.0 to 29.9 kg/m^2^) for cross-sectional analysis during young adulthood and longitudinal analysis.

### Health behaviors and weight status by ethnicity/race and socioeconomic status

Comparison of health behaviors and BMI revealed that they significantly differed by ethnicity/race. White participants had lower screen time, higher MVPA, more frequent breakfast intake, lower SSB intake, less frequent fast-food intake, and lower BMI than several BIPOC groups during adolescence and young adulthood (Table 4). Higher adolescent SES background was associated with higher MVPA, higher breakfast intake, lower fast-food intake and lower BMI in both adolescence and young adulthood. Higher adolescent SES was additionally associated with longer sleep duration and lower SSB intake during adolescence (Table 5).

**Table 4:** Health behaviors and weight status (means) by ethnicity/race, among adolescents (EAT 2010) and young adults (EAT 2018)

**Adolescence (EAT 2010)**							
**Outcome**	**Cohen’s d Statistic**	**Asian American**	**Black/African American**	**Latinx/ Hispanic**	**Mixed/ Other**	**White**	***p*-value**
Screen Time (hours/week)	0.23	38.1 ± 23.0^a^	42.4 ± 31.1^b^	34.9 ± 22.5^a^	43.8 ± 29.9^b^	38.8 ± 23.9^a^	** < 0.001**
MVPA (hours/week)	0.32	5.3 ± 4.1^ab^	5.5 ± 5.5^ac^	4.8 ± 3.9^b^	6.2 ± 5.1^ cd^	7.0 ± 4.1^d^	** < 0.001**
Sleep duration (hours/night)	0.09	8.5 ± 1.1^a^	8.5 ± 1.5^a^	8.4 ± 1.2^a^	8.6 ± 1.2^a^	8.6 ± 1.5^a^	0.511
Breakfast intake (days/week)	0.22	4.0 ± 2.3^a^	4.3 ± 3.0^a^	4.0 ± 2.6^a^	4.3 ± 2.7^a^	4.8 ± 2.3^b^	** < 0.001**
Fruit intake (servings/day)	0.25	1.9 ± 1.6^a^	2.3 ± 2.1^b^	2.3 ± 1.8^b^	2.4 ± 2.0^b^	1.9 ± 1.3^a^	** < 0.001**
Vegetable intake (servings/day)	0.13	1.2 ± 1.0^a^	1.3 ± 1.6^ab^	1.4 ± 1.4^b^	1.4 ± 1.3^ab^	1.3 ± 1.0^ab^	0.192
SSB intake (servings/week)	0.43	3.5 ± 4.2^a^	6.9 ± 7.7^b^	5.2 ± 5.5^ cd^	6.0 ± 6.8^bc^	4.7 ± 4.9^d^	** < 0.001**
Fast-Food intake (times/month)	0.46	9.4 ± 11.8^a^	17.4 ± 20.9^b^	11.9 ± 13.0^c^	14.7 ± 15.5^d^	9.6 ± 8.2^ac^	** < 0.001**
BMI-for-age (percentile)	0.20	69.7 ± 25.9^a^	69.3 ± 32.5^a^	72.7 ± 26.1^a^	71.0 ± 28.4^a^	64.0 ± 25.3^b^	**0.004**
**Young Adulthood (EAT 2018)**							
**Outcome**	**Cohen’s d Statistic**	**Asian American**	**Black/African American**	**Latinx/ Hispanic**	**Mixed/ Other**	**White**	***p*** **-value**
Screen Time (hours/week)	0.20	27.4 ± 11.7^a^	24.1 ± 13.9^b^	24.3 ± 11.5^b^	25.2 ± 13.0^b^	25.1 ± 10.3^b^	**0.005**
MVPA (hours/week)	0.26	3.7 ± 3.5^a^	4.2 ± 4.5^ab^	4.4 ± 3.9^bc^	4.8 ± 4.9^ cd^	5.2 ± 3.8^d^	** < 0.001**
Sleep duration (hours/night)	0.14	8.5 ± 1.4^ab^	8.6 ± 2.0^ac^	8.4 ± 1.5^abc^	8.6 ± 1.5^ac^	8.3 ± 1.1^b^	0.128
Breakfast intake (days/week)	0.20	3.3 ± 2.3^a^	3.7 ± 2.6^ab^	4.0 ± 2.4^bc^	3.5 ± 2.3^ac^	4.0 ± 2.3^b^	**0.004**
Fruit intake (servings/day)	0.29	1.7 ± 1.6^a^	1.9 ± 2.0^ab^	2.1 ± 1.8^bc^	2.4 ± 2.3^c^	1.7 ± 1.3^a^	** < 0.001**
Vegetable intake (servings/day)	0.15	2.1 ± 1.6^ab^	2.0 ± 2.0^b^	2.4 ± 1.8^a^	2.1 ± 1.7^ab^	2.2 ± 1.5^ab^	0.163
SSB intake (servings/week)	0.18	3.0 ± 3.7^a^	3.9 ± 5.9^b^	3.9 ± 4.8^b^	3.9 ± 5.2^b^	4.0 ± 4.8^b^	**0.044**
Fast-Food intake (times/month)	0.44	11.2 ± 12.2^a^	19.1 ± 19.2^b^	14.0 ± 12.3^ cd^	15.5 ± 15.0^d^	11.7 ± 10.2^ac^	** < 0.001**
BMI (kg/m^2^)	0.23	27.4 ± 6.0^abc^	27.1 ± 8.3^b^	28.3 ± 6.8^ac^	27.5 ± 7.9^abc^	25.8 ± 5.8^d^	** < 0.001**

ANOVA tests used to examine differences in unadjusted means by ethnicity/race

^abcd^Within each outcome, cells that share a superscript do not significantly differ at *p* < 0.05

Bold values indicate statistical significance at *p* < 0.05

*MVPA* Moderate-to-vigorous physical activity, *SSB* Sugar-sweetened beverage, *BMI* Body mass index

**Table 5:** Health behaviors and weight status (means) by socioeconomic status among adolescents (EAT 2010) and young adults (EAT 2018)

**Adolescence (EAT 2010)**					
**Outcome**	**Cohen’s d Statistic**	**Low SES**	**Middle SES**	**High SES**	***p-value***
Screen Time (hours/week)	0.05	39.3 ± 26.3^a^	40.9 ± 27.0^a^	40.0 ± 24.7^a^	0.594
MVPA (hours/week)	0.24	5.2 ± 4.7^a^	5.8 ± 4.6^b^	6.7 ± 4.2^c^	** < 0.001**
Sleep duration (hours/night)	0.20	8.4 ± 1.3^a^	8.6 ± 1.3^b^	8.6 ± 0.97^b^	** < 0.001**
Breakfast intake (days/week)	0.29	3.9 ± 2.6^a^	4.2 ± 2.6^a^	4.9 ± 2.3^b^	** < 0.001**
Fruit intake (servings/day)	0.06	2.1 ± 1.8^a^	2.2 ± 1.8^a^	2.1 ± 1.4^a^	0.500
Vegetable intake (servings/day)	0.11	1.3 ± 1.3^a^	1.4 ± 1.4^a^	1.4 ± 1.1^a^	0.133
SSB intake (servings/week)	0.25	6.0 ± 6.5^a^	5.5 ± 6.2^a^	4.0 ± 4.5^b^	** < 0.001**
Fast-Food intake (times/month)	0.16	13.6 ± 16.6^a^	13.5 ± 15.3^a^	10.6 ± 9.5^b^	**0.008**
BMI-for-age (percentile)	0.31	72.3 ± 28.1^a^	70.6 ± 26.5^a^	60.9 ± 27.6^b^	** < 0.001**
**Young Adulthood (EAT 2018)**					
**Outcome**	**Cohen’s d Statistic**	**Low SES**	**Middle SES**	**High SES**	***p*-value**
Screen Time (hours/week)	0.09	25.5 ± 13.0^a^	25.6 ± 12.1^a^	24.3 ± 10.3^a^	0.249
MVPA (hours/week)	0.20	4.0 ± 4.1^a^	4.5 ± 4.2^a^	5.1 ± 3.9^b^	** < 0.001**
Sleep duration (hours/night)	0.11	8.5 ± 1.6^a^	8.5 ± 1.6^a^	8.3 ± 1.2^a^	0.132
Breakfast intake (days/week)	0.17	3.6 ± 2.4^a^	3.7 ± 2.4^a^	4.1 ± 2.2^b^	**0.004**
Fruit intake (servings/day)	0.09	2.0 ± 1.9^a^	2.0 ± 1.8^a^	1.8 ± 1.6^a^	0.276
Vegetable intake (servings/day)	0.12	2.0 ± 1.7^a^	2.2 ± 1.8^a^	2.2 ± 1.6^a^	0.115
SSB intake (servings/week)	0.06	3.9 ± 5.1^a^	3.7 ± 4.8^a^	3.6 ± 4.6^a^	0.579
Fast-Food intake (times/month)	0.20	16.0 ± 16.5^a^	14.7 ± 14.5^a^	12.1 ± 10.0^b^	** < 0.001**
BMI (kg/m^2^)	0.34	28.2 ± 7.7^a^	27.2 ± 6.7^b^	25.1 ± 5.7^c^	** < 0.001**

ANOVA tests used to examine differences in unadjusted means by socioeconomic status

^abc^Within each outcome, cells that share a superscript do not significantly differ at *p* < 0.05

Bold values indicate statistical significance at *p* < 0.05

*SES* Socioeconomic status, *MVPA* Moderate-to-vigorous physical activity, *SSB* Sugar-sweetened beverage, *BMI* Body mass index

Adding interaction terms to fully adjusted regression models provided evidence that ethnicity/race and adolescent SES operated as effect modifiers in the relationship between weight teasing and health behaviors, but only for a few behaviors (approximately 17% of interaction tests). For those behaviors (interaction term with *p* < 0.1), stratified models were examined, and results are displayed in Table 6. For example, in Black/African American adolescents, experiencing weight teasing was cross-sectionally associated with 1.4 fewer hours per week of MVPA (d = 0.30, *p* = 0.012) and 0.7 fewer hours per night of sleep (d = 0.42, *p* = 0.003) versus no significant differences for white adolescents. For young adults from middle SES backgrounds, weight teasing was associated with 0.9 fewer hours per week of MVPA (d = 0.22, *p* = 0.009) and less frequent breakfast intake (teased: 3.2 times per week, compared to not teased: 4.0 times per week, d = 0.31, *p* < 0.001) versus no difference in both measures for young adults from high SES backgrounds.

**Table 6:** Relationships of health behaviors by weight teasing status in young people, effect sizes and marginal means, for variables with evidence of effect modification by ethnicity/race or socioeconomic status, stratified models

Outcome	Cohen’s d Statistic	Marginal Mean (95% CI)		*p*-value	Cohen’s d Statistic	Marginal Mean (95% CI)		*p*-value
		Not Teased	Teased			Not Teased	Teased	
	Black/African American				White			
EAT 2010 Cross-Sectional								
MVPA (hours/week)	0.30	6.0 (5.4, 6.6)	4.6 (3.6, 5.5)	**0.012**	0.14	6.8 (6.2, 7.4)	7.5 (6.7, 8.4)	0.181
EAT 2018 Cross-Sectional								
Sleep duration (hours/night)	0.42	8.9 (8.6, 9.1)	8.2 (7.9, 8.5)	**0.003**	0.02	8.3 (8.2, 8.5)	8.3 (8.1, 8.5)	0.884
Longitudinal (EAT 2010–2018)								
Screen Time (hours/week)	0.30	25.2 (23.7, 26.8)	21.3 (18.8, 23.7)	**0.009**	0.06	24.8 (23.4, 26.2)	25.5 (23.5, 27.5)	0.592
Vegetable intake (servings/day)	0.21	1.9 (1.6, 2.2)	2.3 (1.8, 2.7)	0.128	0.19	2.3 (2.1, 2.6)	2.0 (1.7, 2.3)	0.106
SSB intake (servings/week)	0.18	4.0 (3.2, 4.8)	3.0 (1.7 4.3)	0.188	0.16	3.7 (2.9, 4.4)	4.6 (3.5, 5.6)	0.161
	Asian American				White			
EAT 2010 Cross-Sectional								
Vegetable intake (servings/day)	0.11	1.3 (1.3, 1.5)	1.2 (1.0, 1.4)	0.326	0.26	1.2 (1.1, 1.4)	1.6 (1.3, 1.8)	**0.020**
	Middle SES				High SES			
EAT 2018 Cross-Sectional								
MVPA (hours/week)	0.22	4.9 (4.5, 5.4)	4.0 (3.4, 4.5)	**0.009**	0.08	5.0 (4.4, 5.6)	5.4 (4.7, 6.1)	0.289
Breakfast intake (days/week)^a^	0.31	4.0 (3.7, 4.2)	3.2 (2.9, 3.5)	** < 0.001**	0.14	4.3 (4.0, 4.6)	3.9 (3.5, 4.3)	0.186
Fast-Food intake (times/month)	0.18	14.8 (13.0, 16.6)	17.6 (15.6, 19.6)	0.258	0.38	12.2 (10.7, 13.7)	17.7 (15.9, 19.6)	** < 0.001**

^a^Low SES results: d = 0.05, Not Teased: 3.5 (3.2, 3.8); Teased: 3.6 (3.3, 3.9); *p* = 0.548

Interaction terms were added to the fully adjusted models to test for effect modification by ethnicity/race and SES. If interaction terms were statistically significant (*p* < 0.1), stratified models were examined and results are displayed here

Cross-sectional models adjusted for ethnicity/race, socioeconomic status, gender, and weight status

Longitudinal models adjusted for ethnicity/race, socioeconomic status, gender, outcome assessed at baseline, and weight status

Gender assessed at follow-up; other covariates assessed at baseline. Weighted analyses

Bold values indicate statistical significance at *p* < 0.05

*SES* Socioeconomic status, *MVPA* Moderate-to-vigorous physical activity, *SSB* Sugar-sweetened beverage

## Discussion

In an ethnically/racially and socioeconomically diverse population-based sample of young people, the experience of weight teasing was common and was associated with higher BMI cross-sectionally during both adolescence and young adulthood, and longitudinally over an eight-year follow-up. Weight teasing was cross-sectionally associated with some adverse health behaviors (e.g., shorter sleep duration, less frequent breakfast intake, higher SSB and fast-food intake, longer screen time), although these relationships did not persist longitudinally. BIPOC young people and those from low SES backgrounds had higher weight status, lower MVPA and breakfast frequency, and higher fast-food intake than their respective counterparts. Despite some evidence of effect modification by ethnicity/race and adolescent SES in the weight teasing-health behavior relationship for a minority of behaviors, we did not observe patterns across subgroups or behaviors that suggest weight teasing is more or less harmful for any specific group or outcome. In general, effect sizes were small, although when measuring effects at a population-level, even relatively small differences between exposed and unexposed groups can be important to health outcomes. Results indicate that experiencing weight teasing is cross-sectionally associated with some concerning health behaviors and is a risk factor for future weight gain across ethnic/racial and socioeconomic groups of young people. Overall, these findings provide evidence against potential hypotheses that being exposed to weight teasing might serve as a motivator toward positive changes in eating and activity behaviors.

Findings that weight teasing was associated with higher BMI cross-sectionally during adolescence, cross-sectionally during young adulthood, and longitudinally are consistent with our hypothesis and previous literature [25, 31–34]. Because of our large, diverse study population and longitudinal design, findings strengthen previous evidence that weight stigma is a risk factor for elevated weight status. To our knowledge, this study is the first in adolescents and young adults to examine the relationship between weight stigma and a comprehensive set of weight-related health behaviors. Some studies in young people have examined the relationship between weight stigma and individual health behaviors. For example, a 2019 study by Puhl, Himmelstein, and Watson found that weight teasing from family – but not peers – was associated with difficulty falling asleep [38]. That study population was limited to sexual and gender minorities which may have affected the findings in that youth may have experienced the intersectional effects of weight stigma combined with homophobia and/or transphobia. In comparison, our study found that weight teasing was cross-sectionally associated with shorter sleep duration during both adolescence and young adulthood, after accounting for ethnicity/race, adolescent SES, gender, and weight status. Also, in our measure of weight teasing, we did not assess the teasing source. Further, our study also revealed a cross-sectional relationship between weight teasing and shorter sleep duration in Black/African American adolescents, versus no relationship in white adolescents. These findings and those of Puhl, Himmelstein, and Watson [38] might suggest that intersectionality plays a role in the relationship between weight-based mistreatment and sleep patterns for young people with marginalized identities. However, in our study, there were no differences across ethnic/racial and adolescent SES subgroups in the longitudinal relationship between weight teasing and sleep duration or the cross-sectional relationship in young adulthood.

In addition to shorter sleep duration, weight teasing was cross-sectionally associated with longer screen time during adolescence; cross-sectionally associated with less frequent breakfast intake, higher SSB intake, higher fast-food intake during young adulthood; and longitudinally associated with higher BMI in this sample. These findings align with Tomiyama’s COBWEBS (Cyclic Obesity/Weight-Based Stigma) model, which proposes a “positive feedback loop” between weight-stigmatizing experiences and weight gain [79]. Specifically, Tomiyama postulates that weight-stigmatizing experiences lead to increased stress, which leads to increased cortisol and eating/activity behaviors that promote weight gain, which together lead to elevated weight status, which leads to further weight-stigmatizing experiences [79]. While several prior studies, including our own, have linked weight-stigmatizing experiences to disordered eating behaviors [15, 24, 25], and such behaviors are established risk factors for subsequent weight gain [80–82], the present study may provide evidence for the link between weight-stigmatizing experiences and a different group of eating/activity behaviors that may promote weight gain: shorter sleep duration, longer screen time, less frequent breakfast intake, higher SSB intake, and higher fast-food intake. However, effect sizes were small and there was no evidence of a longitudinal relationship between weight teasing and these behaviors.

Evidence from our study should be interpreted within the context of its strengths and limitations. Strengths include the large, ethnically/racially and socioeconomically diverse sample, allowing us to examine our key research questions through a health equity lens which is important given inequities in numerous nutrition-, fitness- and weight-related health outcomes in young people [4, 41, 42]. Also, pilot testing was a key strength of this study as it allowed the survey items to be phrased in a way that met the developmental needs of participants. Despite its strengths, this study also had several limitations. A single survey item was used to assess some measures (e.g., weight teasing), and it is optimal to use more comprehensive measures. At follow up, height and weight were self-reported, which may have introduced recall or other types of bias. Further, gender/sex was assessed as “male,” “female,” and “different identity.” A more appropriate assessment of these constructs includes the two-question method of birth-assigned sex and gender identity [83]. We did not collect data on sexual orientation, which is a shortcoming, given our health equity lens and our interest in understanding the potential for intersectional impacts of weight stigma. Attrition occurred at follow-up, however, analyses used inverse probability weighting which allowed for extrapolation back to the original sample based on characteristics associated with missingness at follow-up (e.g., lower education of parent/guardian, being born outside the U.S., and identifying as BIPOC). Finally, baseline recruitment took place in one geographic area in Minnesota, U.S., which may limit generalizability of the findings.

## Conclusions

Our study found that weight teasing was cross-sectionally, but not longitudinally, associated with shorter sleep duration, longer screen time, less frequent breakfast intake, higher fast-food intake, and higher SSB intake in an ethnically/racially and socioeconomically diverse sample of young people. The finding that weight teasing was cross-sectionally and longitudinally associated with higher BMI extends previous evidence that weight teasing is both a more common experience for people with high weight and a longitudinal risk factor for weight gain. BIPOC young people and those from low SES backgrounds were disproportionately affected by high weight status, low MVPA, low breakfast intake, and high fast-food intake, and young people across ethnic/racial and adolescent SES subgroups experienced similar effects of weight teasing. When taken together, our findings suggest that clinicians, community-based organizations, public health practitioners, and researchers should view weight stigma as a distinct obstacle to their efforts to address weight-related health in diverse populations of young people. To this end, recommendations have been published and include: 1) increasing awareness of one’s own weight-related biases [7, 84]; 2) avoiding narratives that emphasize personal responsibility in addressing obesity [7, 84]; 3) improving training in strengths-based approaches (e.g., for clinicians, motivational interviewing training) [7, 73]; 4) making the environment of youth-serving clinics and organizations more welcoming for people diverse in body size, ethnicity, race, and SES [7, 73]; 5) reflecting diversity in advertising, media, and brochures, including respectful images of people with high weight [7, 73]; 6) strengthening anti-bullying policies in schools to protect youth from weight-based teasing and bullying [7]; 7) participating in broader advocacy for legislative protections to address body size discrimination [7]; and 8) engaging youth in these efforts [73]. Our findings underscore the importance of these recommendations and the need for increased attention to stigma-reduction efforts. As an initial priority, training for healthcare providers on this topic can foster more compassionate patient care for young people diverse in body size, ethnicity, race, and socioeconomic status; better equip them with tools to address the harms of weight stigma; and create a positive environment to promote health-supporting behaviors.

## Supplementary Information

**Additional file 1:** **Table A.** Cross-sectional relationships of health behaviors and weight status by weight teasing status: means at baseline (EAT 2010, adolescence). **Table B.** Cross-sectional relationships of health behaviors and weight status by weight teasing status: means at follow-up (EAT 2018, young adulthood). **Table C.** Longitudinal relationships of physical activity, nutrition habits, and weight status at 8-year follow-up by weight teasing status at baseline: means (EAT 2010-2018, from adolescence to young adulthood).

## Data Availability

Investigators interested in utilizing the dataset used in the current study should contact the corresponding author. Additional File 1_WT-Bhvrs_3-1–22.doc contains results of two additional main effects models. In Additional File 1_WT-Bhvrs_3-1–22.doc, Model 1 is unadjusted, and Model 2 is adjusted for ethnicity/race, socioeconomic status, and gender.

## Abbreviations

U.S.: United States
BIPOC: Black, Indigenous, and other people of color
SSB: Sugar-sweetened beverages
BMI: Body mass index
SES: Socioeconomic status
FFQ: Food Frequency Questionnaire
YAQ: Young Adult Food Frequency Questionnaire
MVPA: Moderate-to-vigorous physical activity

## References

[1] Ogden CL, Carroll MD, Fryar CD, Flegal KM. Prevalence of Obesity Among Adults and Youth: United States, 2011–2014. NCHS Data Brief. 2015;(219):1–8. Available from: http://www.ncbi.nlm.nih.gov/pubmed/26633046 26633046

[2] Overweight and Obesity. Centers for Disease Control, National Center for Health Statistics. 2016 [cited 4 May 2019]. Available from: https://www.cdc.gov/nchs/fastats/obesity-overweight.htm

[3] Lobstein T, Jackson-Leach R (2006). Estimated burden of paediatric obesity and co-morbidities in Europe. Part 2. Numbers of children with indicators of obesity-related disease. Int J Pediatr Obes.

[4] Trinh-Shevrin C, Islam NS, Nadkarni S, Park R, Kwon SC (2015). Defining an integrative approach for health promotion and disease prevention: a population health equity framework. J Health Care Poor Underserved.

[5] Aaron DG, Stanford FC. Is obesity a manifestation of systemic racism? A ten-point strategy for study and intervention. J Intern Med. 2021 [cited 3 Jul 2021]. 10.1111/joim.13270 10.1111/joim.13270 PMC9908368 33675581

[6] Byrd AS, Toth AT, Stanford FC (2018). Racial disparities in obesity treatment. Curr Obes Rep.

[7] Pont SJ, Puhl R, Cook SR, Slusser W (2017). Stigma experienced by children and adolescents with obesity. Pediatrics..

[8] Pearl RL (2018). Weight bias and stigma: public health implications and structural solutions. Soc Issues Policy Rev.

[9] Puhl RM, Lessard LM (2020). Weight stigma in youth: prevalence, consequences, and considerations for clinical practice. Curr Obes Rep.

[10] Bucchianeri MM, Gower AL, McMorris BJ, Eisenberg ME (2016). Youth experiences with multiple types of prejudice-based harassment. J Adolesc.

[11] Juvonen J, Lessard LM, Schacter HL, Suchilt L (2017). Emotional implications of weight stigma across middle school: the role of weight-based peer discrimination. J Clin Child Adolesc Psychol.

[12] Crenshaw K. Demarginalizing the Intersection of Race and Sex: A Black Feminist Critique of Antidiscrimination Doctrine, Feminist Theory and Antiracist Politics Recommended Citation Crenshaw, Kimberle () "Demarginalizing the Intersection of Race and Sex: A Black Femin. University of Chicago Legal Forum. 1989 [cited 11 Mar 2021]. Available from: http://chicagounbound.uchicago.edu/uclf/vol1989/iss1/8

[13] Himmelstein MS, Puhl RM, Quinn DM (2017). Intersectionality: an understudied framework for addressing weight stigma. Am J Prev Med.

[14] Emmer C, Bosnjak M, Mata J. The association between weight stigma and mental health: a meta-analysis. Obes Rev. 2020 [cited 9 Jul 2020];21(1). Available from: 10.1111/obr.12935%4010.1111/%28ISSN%291758-8111.World_Obesity_Day2020 10.1111/obr.12935 31507062

[15] Hooper L, Puhl R, Eisenberg ME, Crow S, Neumark-Sztainer D. Weight teasing experienced during adolescence and young adulthood: Cross-sectional and longitudinal associations with disordered eating behaviors in an ethnically/racially and socioeconomically diverse sample. Int J Eat Disord. 2021 [cited 10 June 2021]10.1002/eat.23534 10.1002/eat.23534 PMC8355094 33969902

[16] Van Den Berg P, Neumark-Sztainer D, Eisenberg ME, Haines J. Racial/ethnic differences in weight-related teasing in adolescents. Obesity. 2008 [cited 22 Nov 2020];16(SUPPL. 2). Available from: https://pubmed-ncbi-nlm-nih-gov.ezp1.lib.umn.edu/18978760/10.1038/oby.2008.445 18978760

[17] Eisenberg ME, Puhl R, Areba EM, Neumark-Sztainer D (2019). Family weight teasing, ethnicity and acculturation: Associations with well-being among Latinx, Hmong, and Somali Adolescents. J Psychosom Res.

[18] Bucchianeri MM, Eisenberg ME, Wall MM, Piran N, Neumark-Sztainer D (2014). Multiple types of harassment: Associations with emotional well-being and unhealthy behaviors in adolescents. J Adolesc Heal.

[19] Ievers-Landis CE, Dykstra C, Uli N, O’riordan MA (2019). Weight-related teasing of adolescents who are primarily obese: Roles of sociocultural attitudes towards appearance and physical activity self-efficacy. Int J Environ Res Public Health..

[20] Greenleaf C, Petrie TA, Martin SB (2014). Relationship of weight-based teasing and adolescents’ psychological well-being and physical health. J Sch Health.

[21] Puhl RM, Himmelstein MS, Watson RJ (2019). Weight-based victimization among sexual and gender minority adolescents: implications for substance use and mental health. Heal Psychol.

[22] Vartanian LR, Shaprow JG (2008). Effects of weight stigma on exercise motivation and behavior: a preliminary investigation among college-aged females. J Health Psychol.

[23] Haines J, Neumark-Sztainer D, Eisenberg ME, Hannan PJ (2006). Weight teasing and disordered eating behaviors in adolescents: Longitudinal findings from project EAT (Eating Among Teens). Pediatrics..

[24] Hunger JM, Tomiyama AJ (2018). Weight labeling and disordered eating among adolescent girls: longitudinal evidence from the national heart, lung, and blood institute growth and health study. J Adolesc Heal..

[25] Puhl RM, Wall MM, Chen C, Bryn Austin S, Eisenberg ME, Neumark-Sztainer D (2017). Experiences of weight teasing in adolescence and weight-related outcomes in adulthood: A 15-year longitudinal study. Prev Med (Baltim)..

[26] Najjar RH, Jacob E, Evangelista L (2018). Eating behaviors, weight bias, and psychological functioning in multi-ethnic low-income adolescents. J Pediatr Nurs..

[27] Carr D, Friedman MA (2006). Body weight and the quality of interpersonal relationships. Soc Psychol Q.

[28] Puhl RM, Luedicke J (2012). Weight-based victimization among adolescents in the school setting: emotional reactions and coping behaviors. J Youth Adolesc.

[29] Friedman KE, Reichmann SK, Costanzo PR, Zelli A, Ashmore JA, Musante GJ, Weight stigmatization and ideological (2005). Beliefs: relation to psychological functioning in obese adults. Obes Res.

[30] Myers A, Rosen JC (1999). Obesity stigmatization and coping: Relation to mental health symptoms, body image, and self-esteem. Int J Obes..

[31] Haines J, Neumark-Sztainer D, Wall M, Story M (2007). Personal, behavioral, and environmental risk and protective factors for adolescent overweight. Obesity.

[32] Quick V, Wall M, Larson N, Haines J, Neumark-Sztainer D (2013). Personal, behavioral and socio-environmental predictors of overweight incidence in young adults: 10-yr longitudinal findings. Int J Behav Nutr Phys Act.

[33] Schvey NA, Marwitz SE, Mi SJ, Galescu OA, Broadney MM, Young-Hyman D (2019). Weight-based teasing is associated with gain in BMI and fat mass among children and adolescents at-risk for obesity: a longitudinal study. Pediatr Obes.

[34] Hunger JM, Tomiyama AJ (2014). Weight labeling and obesity: a longitudinal study of girls aged 10 to 19 years. JAMA Pediatr.

[35] Foltz JL, Cook SR, Sziliagyi PG, Auinger P, Stewart PA, Bucher S (2011). US adolescent nutrition, exercise, and screen time baseline levels prior to national recommendations. Clin Pediatr (Phila).

[36] Pearl RL, Wadden TA, Jakicic JM (2021). Is weight stigma associated with physical activity? A systematic review. Obesity.

[37] Watanabe PI, Fontana FE, da Silva MP, Mazzardo O, Bacil EDA, de Campos W (2017). Associação entre a provocação referente ao peso corporal e a atividade física em adolescentes. Rev Paul Pediatr.

[38] Puhl RM, Himmelstein MS, Watson RJ (2019). Weight-based victimization among sexual and gender minority adolescents: Findings from a diverse national sample. Pediatr Obes..

[39] Schvey NA, Puhl RM, Brownell KD (2011). The impact of weight stigma on caloric consumption. Obesity.

[40] Sutin A, Robinson E, Daly M, Terracciano A (2016). Weight discrimination and unhealthy eating-related behaviors. Appetite.

[41] Greves Grow HM, Cook AJ, Arterburn DE, Saelens BE, Drewnowski A, Lozano P (2010). Child obesity associated with social disadvantage of children’s neighborhoods. Soc Sci Med.

[42] Spanakis EK, Golden SH (2013). Race/ethnic difference in diabetes and diabetic complications. Curr Diab Rep.

[43] Morgan Hughey S, Kaczynski AT, Child S, Moore JB, Porter D, Hibbert J. Green and lean: Is neighborhood park and playground availability associated with youth obesity? Variations by gender, socioeconomic status, and race/ethnicity. Prev Med (Baltim). 2017;95 Suppl:S101–8. Available from: https://pubmed-ncbi-nlm-nih-gov.ezp2.lib.umn.edu/27932053/10.1016/j.ypmed.2016.11.024 27932053

[44] Hasson RE (2018). Addressing disparities in physical activity participation among African American and Latino Youth. Kinesiol Rev.

[45] Monsivais P, Mclain J, Drewnowski A (2010). The rising disparity in the price of healthful foods: 2004–2008. Food Policy.

[46] Adeigbe RT, Baldwin S, Gallion K, Grier S, Ramirez AG (2015). Food and beverage marketing to Latinos: a systematic literature review. Heal Educ Behav.

[47] Hoffman J. Farm Subsidies Overwhelmingly Support White Farmers. Colorlines. 2009 Jan 29 [cited 8 Feb 2022]; Available from: https://www.colorlines.com/articles/farm-subsidies-overwhelmingly-support-white-farmers

[48] Reynolds BJ. Black Farmers in America, 1865–2000 The Pursuit of Independent Farming and the Role of Cooperatives: RBS Research Report 194. 2002. Available from: https://www.rd.usda.gov/files/RR194.pdf

[49] Larson NI, Wall MM, Story MT, Neumark-Sztainer DR (2013). Home/family, peer, school, and neighborhood correlates of obesity in adolescents. Obesity.

[50] Neumark-Sztainer D, Wall MM, Larson N, Story M, Fulkerson JA, Eisenberg ME (2012). Secular trends in weight status and weight-related attitudes and behaviors in adolescents from 1999 to 2010. Prev Med (Baltim).

[51] Yuan C, Spiegelman D, Rimm EB, Rosner BA, Stampfer MJ, Barnett JB (2017). Validity of a dietary questionnaire assessed by comparison with multiple weighed dietary records or 24-hour recalls. Am J Epidemiol.

[52] Rimm EB, Giovannucci EL, Stampfer MJ, Colditz GA, Litin LB, Willett WC (1992). Reproducibility and validity of an expanded self-administered semiquantitative food frequency questionnaire among male health professionals. Am J Epidemiol.

[53] Feskanich D, Rimm EB, Giovannucci EL, Colditz GA, Stampfer MJ, Litin LB (1993). Reproducibility and validity of food intake measurements from a semiquantitative food frequency questionnaire. J Am Diet Assoc.

[54] Rockett HRH, Breitenbach M, Frazier AL, Witschi J, Wolf AM, Field AE (1997). Validation of a youth/adolescent food frequency questionnaire. Prev Med (Baltim).

[55] Borradaile KE, Foster GD, May H, Karpyn A, Sherman S, Grundy K (2008). Associations between the Youth/Adolescent Questionnaire, the Youth/Adolescent Activity Questionnaire, and body mass index z score in low-income inner-city fourth through sixth grade children. Am J Clin Nutr.

[56] Neumark-Sztainer D, Wall MM, Story M, Perry CL (2003). Correlates of unhealthy weight-control behaviors among adolescents: Implications for prevention programs. Heal Psychol.

[57] Utter J, Neumark-Sztainer D, Jeffery R, Story M (2003). Couch potatoes or French fries: are sedentary behaviors associated with body mass index, physical activity, and dietary behaviors among adolescents?. J Am Diet Assoc.

[58] Godin G, Shephard RJ (1985). A simple method to assess exercise behavior in the community. Can J Appl Sport Sci.

[59] Sirard JR, Hannan P, Cutler GJ, Nuemark-Sztainer D (2013). Evaluation of 2 self-report measures of physical activity with accelerometry in young adults. J Phys Act Heal.

[60] Sirard JR, Bruening M, Wall MM, Eisenberg ME, Kim SK, Neumark-Sztainer D (2013). Physical activity and screen time in adolescents and their friends. Am J Prev Med.

[61] Watts AW, Miller J, Larson NI, Eisenberg ME, Story MT, Neumark-Sztainer D (2018). Multicontextual correlates of adolescent sugar-sweetened beverage intake. Eat Behav.

[62] Bruening M, Eisenberg M, MacLehose R, Nanney MS, Story M, Neumark-Sztainer D (2012). Relationship between adolescents’ and their friends’ eating behaviors: breakfast, fruit, vegetable, whole-grain, and dairy intake. J Acad Nutr Diet.

[63] Bruening M, MacLehose R, Eisenberg ME, Nanney MS, Story M, Neumark-Sztainer D (2014). Associations between sugar-sweetened beverage consumption and fast-food restaurant frequency among adolescents and their friends. J Nutr Educ Behav.

[64] Gibson RS (1990). Principles of nutritional assessment.

[65] Himes JH, Hannan P, Wall M, Neumark-Sztainer D (2005). Factors associated with errors in self-reports of stature, weight, and body mass index in Minnesota adolescents. Ann Epidemiol.

[66] Kuczmarski RJ, Ogden CL, Grummer-Strawn LM, Flegal KM, Guo SS, Wei R (2000). CDC growth charts: United States. Adv Data.

[67] Neumark-Sztainer D, Croll J, Story M, Hannan PJ, French SA, Perry C (2002). Ethnic/racial differences in weight-related concerns and behaviors among adolescent girls and boys: Findings from Project EAT. J Psychosom Res..

[68] Brieman L, Friedman J, Olshen R, Stone C (1984). Classification and regression trees.

[69] Montez JK, Hayward MD (2014). Cumulative childhood adversity, educational attainment, and active life expectancy among U.S. adults. Demography.

[70] Lown EA, Lui CK, Karriker-Jaffe K, Mulia N, Williams E, Ye Y (2019). Adverse childhood events and risk of diabetes onset in the 1979 National longitudinal survey of youth cohort. BMC Public Health.

[71] Gardner R, Feely A, Layte R, Williams J, McGavock J (2019). Adverse childhood experiences are associated with an increased risk of obesity in early adolescence: a population-based prospective cohort study. Pediatr Res.

[72] Hooper L, Mason SM, Telke S, Larson N, Neumark-Sztainer D. Experiencing Household Food Insecurity During Adolescence Predicts Disordered Eating and Elevated Body Mass Index 8 Years Later. J Adolesc Health. 2022 [cited 12 Apr 2022]; Available from: https://pubmed.ncbi.nlm.nih.gov/35078732/10.1016/j.jadohealth.2021.11.026 PMC9038678 35078732

[73] Svetaz MV, Chulani V, West KJ, Voss R, Kelley MA, Raymond-Flesch M (2018). Racism and its harmful effects on nondominant racial-ethnic youth and youth-serving providers: a call to action for organizational change: the society for adolescent health and medicine. J Adolesc Heal.

[74] Suttorp MM, Siegerink B, Jager KJ, Zoccali C, Dekker FW (2015). NDT perspectives graphical presentation of confounding in directed acyclic graphs. Nephrol Dial Transpl.

[75] Rothman KJ, Lash TLL, Greenland S (2008). Modern Epidemiology.

[76] Cohen J (1988). Statistical Power Analysis for the Behavioral Sciences.

[77] Little RJA. Survey Nonresponse Adjustments for Estimates of Means. Int Stat Rev / Rev Int Stat. 1986;54(2):139. Available from: https://www.jstor.org/stable/1403140

[78] Seaman SR, White IR (2013). Review of inverse probability weighting for dealing with missing data. Stat Methods Med Res.

[79] Tomiyama AJ. Weight stigma is stressful. A review of evidence for the cyclic Obesity/weight-based stigma model. Appetite. 2014;82:8–15. 10.1016/j.appet.2014.06.108 24997407

[80] Yoon C, Mason SM, Hooper L, Eisenberg ME, Neumark-Sztainer D (2019). Disordered eating behaviors and 15-year trajectories in body mass index: findings from project eating and activity in teens and young adults (EAT). J Adolesc Heal.

[81] Sonneville KR, Horton NJ, Micali N, Crosby RD, Swanson SA, Solmi F (2013). Longitudinal associations between binge eating and overeating and adverse outcomes among adolescents and young adults: does loss of control matter?. JAMA Pediatr.

[82] Herpertz-Dahlmann B, Dempfle A, Konrad K, Klasen F, Ravens-Sieberer U (2015). Eating disorder symptoms do not just disappear: the implications of adolescent eating-disordered behaviour for body weight and mental health in young adulthood. Eur Child Adolesc Psychiatry.

[83] GenIUSS Group. Best Practices for Asking Questions to Identify Transgender and Other Gender Minority Respondents on Population-Based Surveys. Los Angeles; 2014 Sep [cited 10 Feb 2022]. Available from: https://williamsinstitute.law.ucla.edu/publications/geniuss-trans-pop-based-survey/

[84] Rubino F, Puhl RM, Cummings DE, Eckel RH, Ryan DH, Mechanick JI (2020). Joint international consensus statement for ending stigma of obesity. Nat Med.

