# Supplementary material for: How is weight teasing cross-sectionally and longitudinally associated with health behaviors and weight status among ethnically/racially and socioeconomically diverse young people?
Source: Int J Behav Nutr Phys Act. 2022 Jun 23;19:71. doi: 10.1186/s12966-022-01307-y (PMC9219184; doi:10.1186/s12966-022-01307-y)
Supplement: Supplementary file 1 — Additional file 1: Table A. Cross-sectional relationships of health behaviors and weight status by weight teasing status: means at baseline (EAT 2010, adolescence). Table B. Cross-sectional relationships of health behaviors and weight status by weight teasing status: means at follow-up (EAT 2018, young adulthood). Table C. Longitudinal relationships of physical activity, nutrition habits, and weight status at 8-year follow-up by weight teasing status at baseline: means (EAT 2010-2018, from adolescence to young adulthood). [file 12966_2022_1307_MOESM1_ESM.docx]

Table A. Cross-sectional relationships of health behaviors and weight status by weight teasing status: means at baseline (EAT 2010, adolescence)

| Outcome | Model 1 | | | | Model 2 | | | |
| --- | --- | --- | --- | --- | --- | --- | --- | --- |
|  | Cohen’s | Mean (95% CI) | | p-value | Cohen’s | Marginal Mean (95% CI) | | p-value |
|  | d  Statistic | Not Teased | Teased |  | d  Statistic | Not Teased | Teased |  |
| Screen Time (hours/week) | 0.10 | 38.9 (37.3, 40.6) | 41.6 (39.3, 43.8) | 0.061 | 0.13 | 39.0 (37.4, 40.6) | 42.4 (40.1, 44.6) | **0.015** |
|  |  |  |  |  |  |  |  |  |
| MVPA (hours/week) | 0.05 | 5.9 (5.6, 6.2) | 5.5 (5.1, 5.9) | 0.087 | 0.06 | 5.9 (5.6, 6.1) | 5.6 (5.2, 6.0) | 0.313 |
|  |  |  |  |  |  |  |  |  |
| Sleep duration (hours/night) | 0.11 | 8.6 (8.5, 8.7) | 8.4 (8.3, 8.5) | **0.035** | 0.12 | 8.6 (8.5, 8.6) | 8.4 (8.3, 8.5) | **0.024** |
|  |  |  |  |  |  |  |  |  |
| Breakfast intake (days/week) | 0.13 | 4.4 (4.2, 4.6) | 4.0 (3.8, 4.3) | **0.011** | 0.13 | 4.4 (4.2, 4.6) | 4.0 (3.8, 4.3) | **0.010** |
|  |  |  |  |  |  |  |  |  |
| Fruit intake (servings/day) | 0.05 | 2.1 (2.0, 2.2) | 2.2 (2.1, 2.4) | 0.336 | 0.07 | 2.1 (2.0, 2.2) | 2.2 (2.1, 2.4) | 0.220 |
|  |  |  |  |  |  |  |  |  |
| Vegetable intake (servings/day) | 0.14 | 1.3 (1.2, 1.3) | 1.5 (1.3, 1.6) | **0.007** | 0.16 | 1.3 (1.2, 1.4) | 1.5 (1.4, 1.6) | **0.003** |
|  |  |  |  |  |  |  |  |  |
| SSB intake (servings/week) | 0.02 | 5.3 (4.9, 5.7) | 5.4 (4.8, 5.9) | 0.781 | 0.00 | 5.4 (5.0, 5.8) | 5.5 (5.0, 6.0) | 0.820 |
|  |  |  |  |  |  |  |  |  |
| Fast-Food intake (times/month) | 0.06 | 12.6 (11.7, 13.6) | 13.6 (12.3, 14.9) | 0.232 | 0.05 | 12.6 (11.8, 13.6) | 13.4 (12.2, 14.7) | 0.350 |
|  |  |  |  |  |  |  |  |  |
| BMI-for-age (percentile) | 0.30 | 66.3 (64.6, 68.0) | 75.0 (72.6, 77.4) | **<0.001** | See Table 2 | | | |

Model 1: unadjusted

Model 2: adjusted for ethnicity/race, socioeconomic status, and gender

Gender assessed at follow-up; other covariates assessed at baseline

Sample size range: 1383-1479; Weighted analyses

MVPA: moderate-to-vigorous physical activity; SSB: sugar-sweetened beverage; BMI: body mass indexTable B. Cross-sectional relationships of health behaviors and weight status by weight teasing status: means at follow-up (EAT 2018, young adulthood)

| Outcome | Model 1 | | | | Model 2 | | | |
| --- | --- | --- | --- | --- | --- | --- | --- | --- |
|  | Cohen’s | Mean (95% CI) | | p-value | Cohen’s | Marginal Mean (95% CI) | | p-value |
|  | d  Statistic | Not Teased | Teased |  | d  Statistic | Not Teased | Teased |  |
| Screen Time (hours/week) | 0.14 | 24.5 (23.7, 25.3) | 26.2 (25.3, 27.2) | **0.007** | 0.11 | 24.8 (23.9, 25.6) | 26.1 (25.2, 27.1) | **0.032** |
|  |  |  |  |  |  |  |  |  |
| MVPA (hours/week) | 0.10 | 4.6 (4.3, 4.8) | 4.2 (3.8, 4.5) | 0.052 | 0.06 | 4.6 (4.3, 4.8) | 4.3 (4.0, 4.6) | 0.236 |
|  |  |  |  |  |  |  |  |  |
| Sleep duration (hours/night) | 0.21 | 8.6 (8.5, 8.7) | 8.3 (8.2, 8.4) | **<0.001** | 0.23 | 8.6 (8.5, 8.7) | 8.3 (8.1, 8.4) | **<0.001** |
|  |  |  |  |  |  |  |  |  |
| Breakfast intake (days/week) | 0.16 | 3.9 (3.7, 4.0) | 3.5 (3.3, 3.7) | **0.002** | 0.13 | 3.8 (3.7, 4.0) | 3.5 (3.3, 3.7) | **0.011** |
|  |  |  |  |  |  |  |  |  |
| Fruit intake (servings/day) | 0.02 | 1.9 (1.8, 2.1) | 1.9 (1.8, 2.1) | 0.759 | 0.03 | 2.0 (1.9, 2.1) | 1.9 (1.8, 2.1) | 0.558 |
|  |  |  |  |  |  |  |  |  |
| Vegetable intake (servings/day) | 0.07 | 2.2 (2.1, 2.3) | 2.1 (1.9, 2.2) | 0.253 | 0.06 | 2.2 (2.1, 2.3) | 2.1 (1.9, 2.2) | 0.337 |
|  |  |  |  |  |  |  |  |  |
| SSB intake (servings/week) | 0.10 | 3.5 (3.2, 3.9) | 4.0 (3.6, 4.5) | 0.083 | 0.13 | 3.5 (3.2, 3.9) | 4.2 (3.7, 4.6) | **0.026** |
|  |  |  |  |  |  |  |  |  |
| Fast-Food intake (times/month) | 0.21 | 13.2 (12.3, 14.2) | 16.3 (15.2, 17.4) | **<0.001** | 0.22 | 13.3 (12.3, 14.2) | 16.5 (15.3, 17.6) | **<0.001** |
|  |  |  |  |  |  |  |  |  |
| BMI (kg/m^2^) | 0.48 | 25.8 (25.4, 26.2) | 29.1 (28.5, 29.6) | **<0.001** | See Table 2 | | | |

Model 1: unadjusted

Model 2: adjusted for ethnicity/race, socioeconomic status, and gender

Gender assessed at follow-up; other covariates assessed at baseline

Sample size range: 1190-1442; Weighted analyses

MVPA: moderate-to-vigorous physical activity; SSB: sugar-sweetened beverage; BMI: body mass index

Table C. Longitudinal relationships of physical activity, nutrition habits, and weight status at 8-year follow-up by weight teasing status at baseline: means (EAT 2010-2018, from adolescence to young adulthood)

| Outcome | Model 1 | | | | Model 2 | | | |
| --- | --- | --- | --- | --- | --- | --- | --- | --- |
|  | Cohen’s | Mean (95% CI) | | p-value | Cohen’s | Marginal Mean (95% CI) | | p-value |
|  | d  Statistic | Not Teased | Teased |  | d  Statistic | Not Teased | Teased |  |
| Screen Time (hours/week) | 0.06 | 24.9 (24.2, 25.7) | 25.7 (24.7, 26.8) | 0.223 | 0.00 | 25.2 (24.4, 25.9) | 25.4 (24.3, 26.4) | 0.789 |
|  |  |  |  |  |  |  |  |  |
| MVPA (hours/week) | 0.02 | 4.4 (4.2, 4.7) | 4.4 (4.0, 4.7) | 0.708 | 0.02 | 4.4 (4.2, 4.7) | 4.5 (4.2, 4.8) | 0.645 |
|  |  |  |  |  |  |  |  |  |
| Sleep duration (hours/night) | 0.08 | 8.5 (8.4, 8.6) | 8.4 (8.3, 8.5) | 0.123 | 0.09 | 8.5 (8.4, 8.6) | 8.4 (8.2, 8.5) | 0.095 |
|  |  |  |  |  |  |  |  |  |
| Breakfast intake (days/week) | 0.00 | 3.7 (3.5, 3.8) | 3.7 (3.5, 3.9) | 0.989 | 0.03 | 3.7 (3.5, 3.8) | 3.8 (3.6, 4.0) | 0.489 |
|  |  |  |  |  |  |  |  |  |
| Fruit intake (servings/day) | 0.02 | 1.9 (1.8, 2.0) | 2.0 (1.8, 2.1) | 0.744 | 0.03 | 1.9 (1.8, 2.1) | 2.0 (1.8, 2.2) | 0.547 |
|  |  |  |  |  |  |  |  |  |
| Vegetable intake (servings/day) | 0.03 | 2.1 (2.0, 2.2) | 2.2 (2.0, 2.3) | 0.562 | 0.05 | 2.1 (2.0, 2.3) | 2.2 (2.1, 2.4) | 0.460 |
|  |  |  |  |  |  |  |  |  |
| SSB intake (servings/week) | 0.06 | 3.6 (3.3, 4.0) | 3.9 (3.4, 4.4) | 0.322 | 0.06 | 3.6 (3.3, 4.0) | 3.9 (3.4, 4.4) | 0.316 |
|  |  |  |  |  |  |  |  |  |
| Fast-Food intake (times/month) | 0.00 | 14.6 (13.7, 15.5) | 14.7 (13.5, 15.9) | 0.937 | 0.02 | 14.5 (13.6, 15.4) | 14.8 (13.6, 16.0) | 0.765 |
|  |  |  |  |  |  |  |  |  |
| BMI-for-age (percentile) | 0.45 | 26.1 (25.6, 26.5) | 29.3 (28.7, 29.9) | **<0.001** | See Table 3 | | | |

Model 1: unadjusted

Model 2: adjusted for ethnicity/race, socioeconomic status, gender, and outcome assessed at baseline

Gender assessed at follow-up; other covariates assessed at baseline.

Sample size range: 1130-1475; Weighted analyses

MVPA: moderate-to-vigorous physical activity; SSB: sugar-sweetened beverage; BMI: body mass index
